# Cytochrome *b*_5_ plays a dual role in the reaction cycle of cytochrome P450 3A4 during oxidation of the anticancer drug ellipticine

**DOI:** 10.1007/s00706-017-1986-9

**Published:** 2017-07-04

**Authors:** Marie Stiborová, Radek Indra, Eva Frei, Kateřina Kopečková, Heinz H. Schmeiser, Tomáš Eckschlager, Vojtěch Adam, Zbyněk Heger, Volker M. Arlt, Václav Martínek

**Affiliations:** 1 Department of Biochemistry, Faculty of Science, Charles University, Albertov 2030, 128 40 Prague 2, Czech Republic; 2 Department of Oncology, 2nd Faculty of Medicine, Charles University and University Hospital Motol, V Uvalu 84, 150 06 Prague 5, Czech Republic; 3 Division of Radiopharmaceutical Chemistry, German Cancer Research Center (DKFZ), Im Neuenheimer Feld 280, 69120 Heidelberg, Germany; 4 Department of Pediatric Hematology and Oncology, 2nd Medical Faculty, Charles University and University Hospital Motol, V Uvalu 84, 150 06 Prague 5, Czech Republic; 5 Laboratory of Metallomics and Nanotechnology, Department of Chemistry and Biochemistry, Mendel University in Brno, Zemedelska 1, 61300 Brno, Czech Republic; 6 Analytical and Environmental Sciences Division, MRC-PHE Centre for Environment and Health, King’s College London, London, SE1 9NH UK

**Keywords:** DNA, Enzymes, Coenzymes, High pressure liquid chromatography

## Abstract

**Abstract:**

Ellipticine is an anticancer agent that forms covalent DNA adducts after enzymatic activation by cytochrome P450 (CYP) enzymes, mainly by CYP3A4. This process is one of the most important ellipticine DNA-damaging mechanisms for its antitumor action. Here, we investigated the efficiencies of human hepatic microsomes and human recombinant CYP3A4 expressed with its reductase, NADPH:CYP oxidoreductase (POR), NADH:cytochrome *b*_5_ reductase and/or cytochrome *b*_5_ in Supersomes™ to oxidize this drug. We also evaluated the effectiveness of coenzymes of two of the microsomal reductases, NADPH as a coenzyme of POR, and NADH as a coenzyme of NADH:cytochrome *b*_5_ reductase, to mediate ellipticine oxidation in these enzyme systems. Using HPLC analysis we detected up to five ellipticine metabolites, which were formed by human hepatic microsomes and human CYP3A4 in the presence of NADPH or NADH. Among ellipticine metabolites, 9-hydroxy-, 12-hydroxy-, and 13-hydroxyellipticine were formed by hepatic microsomes as the major metabolites, while 7-hydroxyellipticine and the ellipticine *N* ^2^-oxide were the minor ones. Human CYP3A4 in Supersomes™ generated only three metabolic products, 9-hydroxy-, 12-hydroxy-, and 13-hydroxyellipticine. Using the ^32^P-postlabeling method two ellipticine-derived DNA adducts were generated by microsomes and the CYP3A4-Supersome system, both in the presence of NADPH and NADH. These adducts were derived from the reaction of 13-hydroxy- and 12-hydroxyellipticine with deoxyguanosine in DNA. In the presence of NADPH or NADH, cytochrome *b*_5_ stimulated the CYP3A4-mediated oxidation of ellipticine, but the stimulation effect differed for individual ellipticine metabolites. This heme protein also stimulated the formation of both ellipticine-DNA adducts. The results demonstrate that cytochrome *b*_5_ plays a dual role in the CYP3A4-catalyzed oxidation of ellipticine: (1) cytochrome *b*_5_ mediates CYP3A4 catalytic activities by donating the first and second electron to this enzyme in its catalytic cycle, indicating that NADH:cytochrome *b*_5_ reductase can substitute NADPH-dependent POR in this enzymatic reaction and (2) cytochrome *b*_5_ can act as an allosteric modifier of the CYP3A4 oxygenase.

**Graphical abstract:**

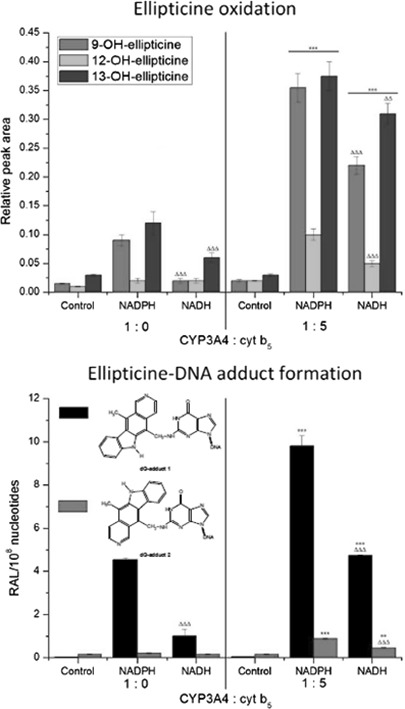

## Introduction

**Fig. 1:**
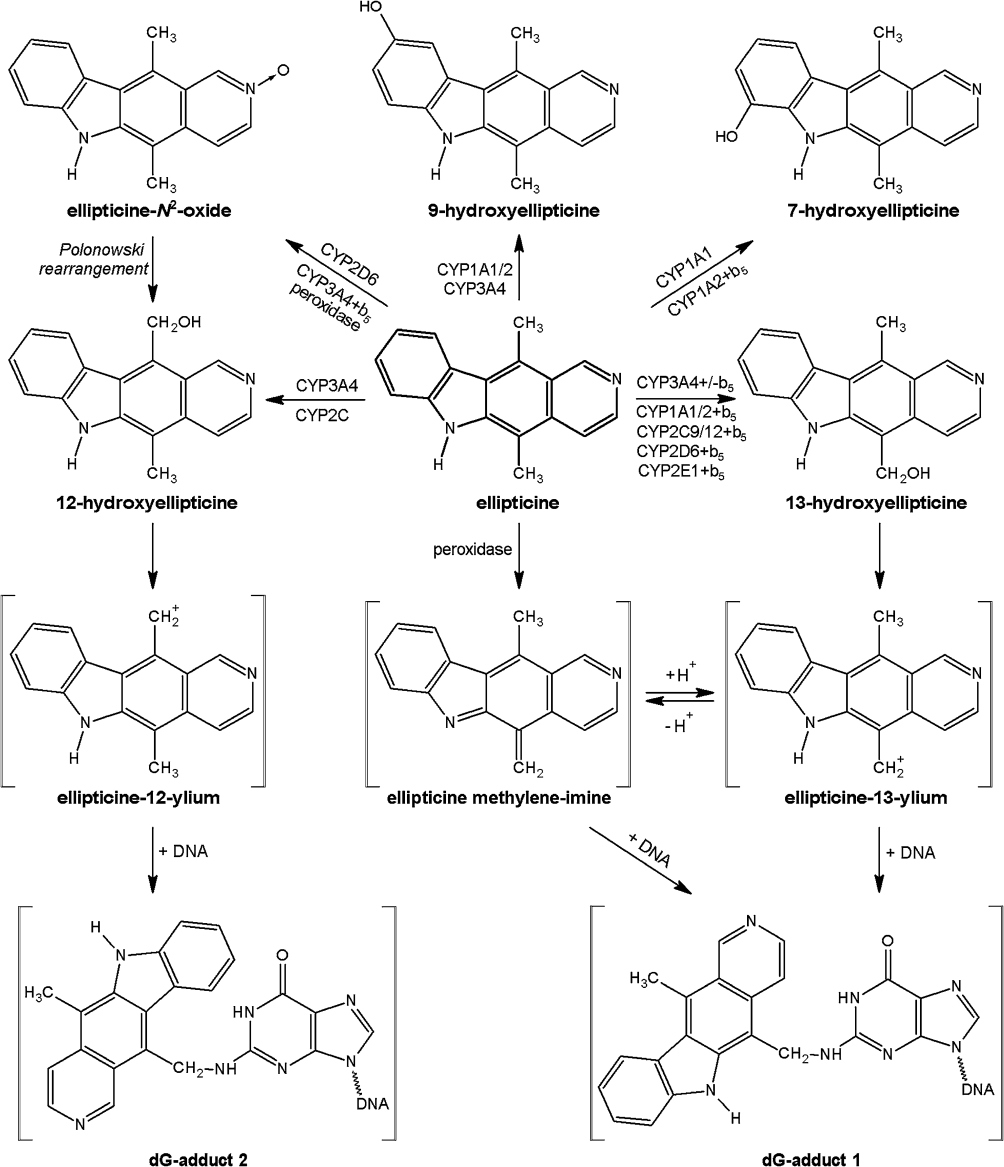
Scheme of ellipticine metabolism by CYPs and peroxidases showing the identified metabolites and those proposed to form DNA adducts. The compounds shown in *brackets* were not detected under the experimental conditions and/or not structurally characterized. The CYP enzymes predominantly oxidizing ellipticine shown in the figure were identified in this work and/or in previous studies [11, 16]

Ellipticine (5,11-dimethyl-6*H*-pyrido[4,3-*b*]carbazole, see Fig. 1) and its derivatives are efficient anticancer compounds that function through multiple mechanisms participating in cell cycle arrest and initiation of apoptosis (for a summary see [1–8]). The predominant mechanisms of ellipticine’s biological effects were proposed to be intercalation into DNA [1, 6, 9] and the inhibition of topoisomerase II [1, 4, 6–8]. Further, we have shown that this antitumor agent forms covalent DNA adducts after enzymatic activation with cytochromes P450 (CYP) and peroxidases [2, 4, 7, 8, 10–17], suggesting an additional DNA-damaging effect of ellipticine. Of the CYP enzymes investigated, human CYP3A4 is the most active enzyme oxidizing ellipticine to 12-hydroxy- and 13-hydroxyellipticine, reactive metabolites that dissociate to ellipticine-12-ylium and ellipticine-13-ylium and bind to DNA (see Fig. 1) [4, 5, 11, 12]. The CYP3A4 isoform also forms other ellipticine metabolites, including 9-hydroxyellipticine, which is a detoxification product, as well as 7-hydroxyellipticine and ellipticine *N* ^2^-oxide as minor metabolites (Fig. 1) [4, 5, 15, 16].

CYP enzymes, including CYP3A4, are components of the monooxygenase system located in the membrane of the endoplasmic reticulum (microsomes). This enzymatic system also contains other enzymes such as NADPH:cytochrome P450 oxidoreductase (POR) and cytochrome *b*_5_ accompanied with its reductase, NADH:cytochrome *b*_5_ reductase. The functions of this system are complex, but a common feature is that, to perform the mono-oxygenation reaction with molecular oxygen and all CYP enzymes involved in the metabolism of xenobiotics receive electrons from POR [18, 19].

The second electron needed for the reduction of CYP in the monooxygenase reaction cycle may also be provided by cytochrome *b*_5_ in the presence of POR or NADH:cytochrome *b*_5_ reductase, but cytochrome *b*_5_ also plays additional roles in the monooxygenase system [15, 17, 20–24]. It was demonstrated that a second electron donor, cytochrome *b*_5_, could also modulate (stimulate and/or inhibit) the activity of several CYPs [21, 25], both in vitro [26–32] and in vivo [19, 24, 33–35]. Cytochrome *b*_5_ has been reported to stimulate the oxidation of a variety of CYP3A4 substrates, including ellipticine [15–17, 19, 26, 29, 36–38]. Interestingly, in the case of ellipticine, cytochrome *b*_5_, when reconstituted with CYP3A4 and POR, alters the ratio of ellipticine metabolites formed by this CYP. Under these conditions, the amounts of the detoxification metabolites (7-hydroxyellipticine and 9-hydroxyellipticine) were not changed with added cytochrome *b*_5_, whereas oxidation of ellipticine to 12-hydroxyellipticine, 13-hydroxyellipticine, and ellipticine *N* ^2^-oxide, the metabolites responsible for the formation of covalent DNA adducts [4, 5, 11–13], increased considerably [15]. Consequently, this led to an increase in the generation of ellipticine-derived DNA adducts. Hence, cytochrome *b*_5_ seems to play a key role in the CYP3A4-mediated DNA-damage caused by these ellipticine metabolites [15, 16]. CYP3A4-mediated oxidation of ellipticine was significantly changed only by holo-cytochrome *b*_5_ containing heme, while neither apo-cytochrome *b*_5_ without heme or Mn-cytochrome *b*_5_ had such an effect [15].

Results found by Guengerich and his coworkers [26, 28] demonstrated that cytochrome *b*_5_ added to recombinant CYP3A4 reconstituted with POR enhanced CYP3A4 marker activity, testosterone 6-β-hydroxylation. They also showed that this heme protein with NADH:cytochrome *b*_5_ reductase and NADH might even substitute the POR/NADPH system in the CYP3A4 catalysis of this marker reaction. Recently, we have found that the NADH:cytochrome *b*_5_ reductase/cytochrome *b*_5_/NADH system can also substitute the POR/NADPH system and act as an electron donor to another CYP enzyme, CYP1A1, for the oxidation of benzo[*a*]pyrene (BaP). This NADH-dependent system can, therefore, function as a sole electron donor for both reduction steps in the reaction cycles of CYP1A1 [39, 40] and CYP3A4 [26, 28]. Here, we investigated in detail, whether the NADH:cytochrome *b*_5_ reductase/cytochrome *b*_5_/NADH system can also be the sole electron donor in the CYP3A4-catalyzed oxidation of ellipticine, Another aim of this work was to shed more light on the further functions of cytochrome *b*_5_ in this CYP3A4-mediated reaction.

## Results and discussion

### Oxidation of ellipticine by human hepatic microsomes and human CYP3A4 expressed in Supersomes™ in the presence of NADPH and NADH

In the last decade, we have studied in detail the CYP-mediated oxidation of ellipticine using the monooxygenase systems of several species founding that up to five oxidized metabolites are formed [3–5, 8, 11, 15–17, 40–42]. Since reactions of CYPs use molecular oxygen, electrons are essential for this process. In addition to the classical reductase POR with its cofactor NADPH, cytochrome *b*_5_ plus NADH:cytochrome *b*_5_ reductase has come into focus, and, therefore, its role in ellipticine oxidation was the aim of this study. Three enzymatic systems were utilized for such a study: (i) human hepatic microsomes, (ii) microsomes of baculovirus-infected insect cells (Supersomes™) containing over-expressed amounts of human recombinant CYP3A4 and POR, as well as the basal levels of NADH:cytochrome *b*_5_ reductase and cytochrome *b*_5_, and (iii) the analogous Supersomes™, but also containing over-expressed cytochrome *b*_5_, at a molar ratio of CYP3A4 to cytochrome *b*_5_ of 1:5. NADPH and NADH, cofactors of the two microsomal reductases POR and NADH:cytochrome *b*_5_ reductase, respectively, were utilized as electron donors for CYP-mediated ellipticine oxidation. The ellipticine metabolite profile formed by the used enzymatic systems was determined by HPLC analysis and ellipticine metabolites were identified by NMR and/or mass spectrometry as described previously [11].

**Fig. 2:**
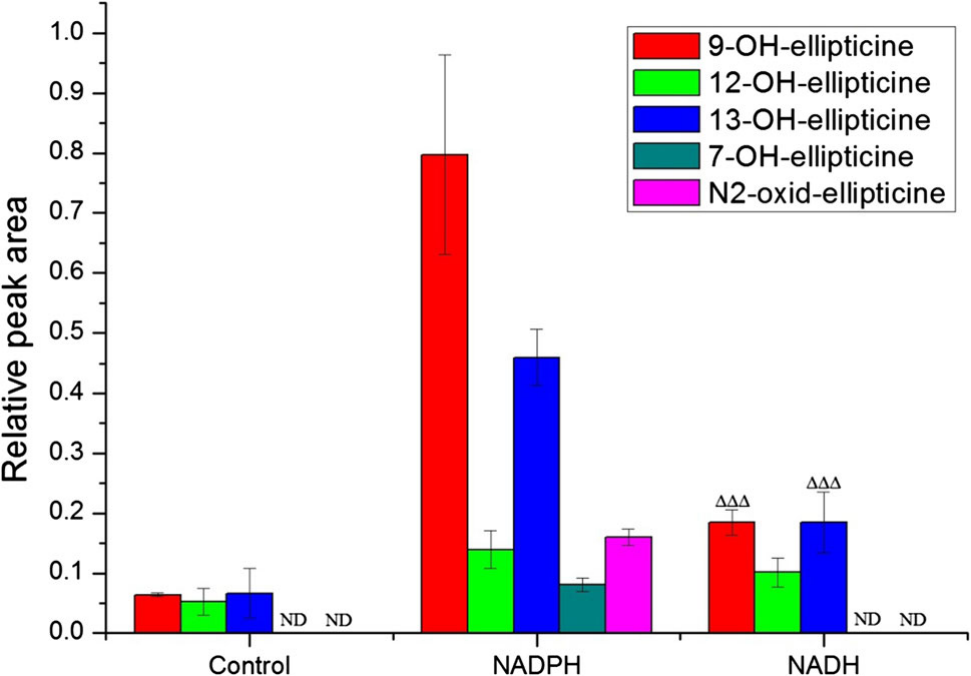
Amounts of ellipticine metabolites generated by human hepatic microsomes in the presence of NADPH or NADH. Data shown are mean peak areas relative to the internal standard phenacetin ± SD (*n* = 3; analyses of three independent in vitro incubations). *ND* not detected, ^∆∆∆^ *P* < 0.001 (Student’s *t* test), significantly different from incubations in the presence of NADPH as a cofactor. Control—incubations without added cofactors

As shown in our previous studies [11, 16], in the presence of NADPH five ellipticine metabolites were formed in human hepatic microsomes. They were structurally characterized previously [11] to be 9-hydroxy-, 12-hydroxy-, 13-hydroxy-, and 7-hydroxyellipticine, in addition to *N* ^2^-oxide of ellipticine. Here, we show that three of these metabolites are also formed in human hepatic microsomes in the presence of NADH instead of NADPH (Fig. 2). As with NADPH, 9-hydroxyellipticine and 13-hydroxyellipticine were produced as the major metabolic products, while 12-hydroxyellipticine was the minor one. 7-Hydroxyellipticine and ellipticine *N* ^2^-oxide were not detectable in the presence of NADH (Fig. 2). In the presence of NADH, the amounts of ellipticine metabolites were up to five-times lower than in the presence of NADPH. Negligible amounts or no ellipticine metabolites (7-hydroxyellipticine and ellipticine *N* ^2^-oxide) were found when NADPH or NADH was omitted from the incubation mixtures containing human hepatic microsomes (Fig. 2).

These results demonstrated that not only NADPH, but also NADH acts as an electron donor for the reduction of CYPs during ellipticine oxidation in human liver microsomes. These results also suggested that NADH can even serve as an exclusive electron donor for CYP in its catalytic cycle independent of NADPH and POR. This suggestion was confirmed by the finding that NADH does not function as a coenzyme of POR when cytochrome *c* is used as a substrate [39, 40].

**Fig. 3:**
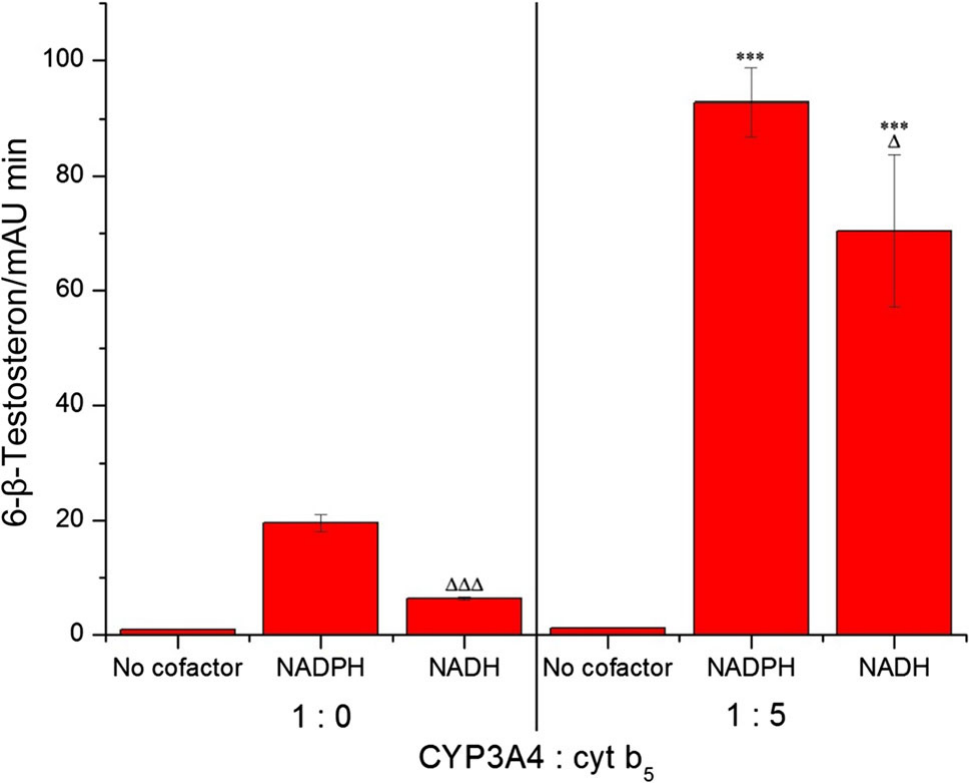
Testosteron 6-β-hydroxylation catalyzed by CYP3A4-Supersomes™ with and without cytochrome *b*_5_ (at a molar ratio of CYP3A4:cytochrome *b*_5_ of 1:5) in the presence of NADPH or NADH. Data shown are mean peak areas relative to the internal standard phenacetin ± SD (*n* = 3; analyses of three independent in vitro incubations). *ND* not detected. ^∆∆∆^ *P* < 0.001, ^∆^ *P* < 0.05 (Student’s *t* test), significantly different from incubations in the presence of NADPH as a cofactor; ***P < 0.001 (Student’s *t* test), significantly different from incubations without cytochrome *b*_5_

To evaluate the suitability of the CYP3A4-Supersomes™ systems [see systems (ii) and (iii)] for our experiments, we first tested whether they are functionally active by testing the CYP3A4 marker activity, testosterone 6-β-hydroxylation. Our results showed that the Supersomal CYP3A4 systems are capable of catalyzing this marker reaction, both in the presence of NADPH and NADH, and cytochrome *b*_5_ stimulated this reaction enormously (Fig. 3). Based on these findings, we could argue that the Supersomal CYP3A4 systems were suitable for our further enzymatic studies, where ellipticine was analyzed to be a CYP3A4 substrate. Our results with these CYP3A4 systems are fully in accordance with the studies by Guengerich and his coworkers [26, 28], where they used human recombinant CYP3A4 reconstituted either with POR plus NADPH or NADH:cytochrome *b*_5_ reductase with NADH in the presence of cytochrome *b*_5_ in liposomes.

**Fig. 4:**
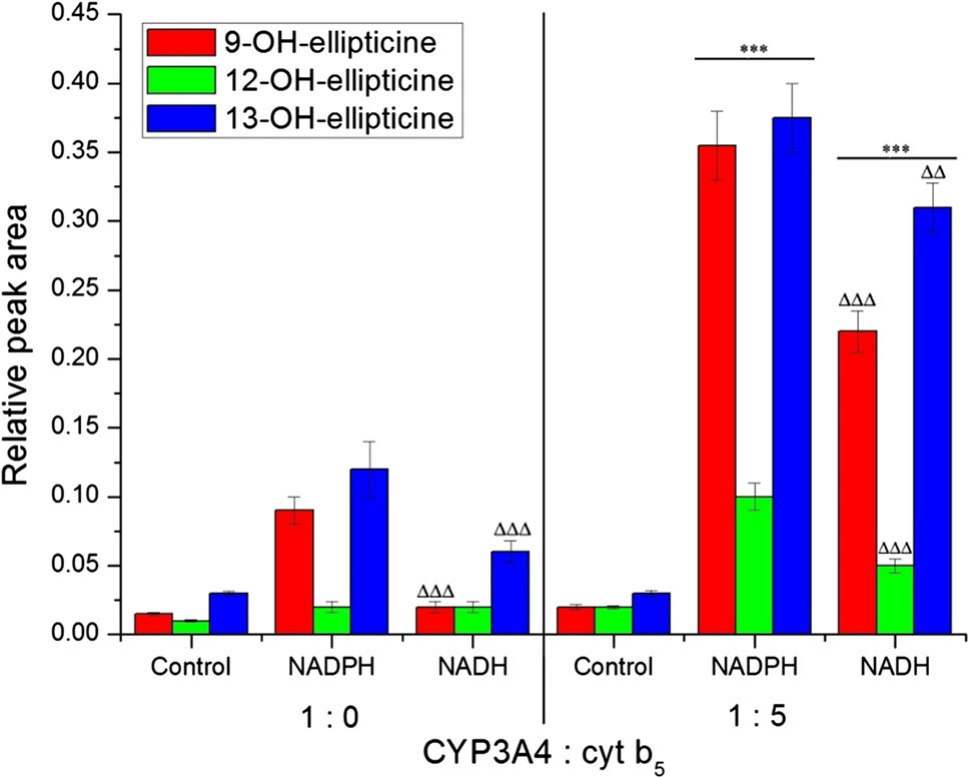
Amounts of ellipticine metabolites generated by human CYP3A4 in the presence of NADPH or NADH with or without cytochrome *b*_5_ (at a molar ratio of CYP3A4:cytochrome *b*_5_ of 1:5). Data shown are mean peak areas relative to the internal standard phenacetin ± SD (*n* = 3; analyses of three independent in vitro incubations). *ND* not detected. ^∆∆∆^ *P* < 0.001, ^∆^ *P* < 0.01 (Student’s *t* test), significantly different from incubations in the presence of NADPH as a cofactor; ***P < 0.001 (Student’s *t* test), significantly different from incubations without cytochrome *b*_5_. Control—incubations without added cofactors

Human recombinant CYP3A4 expressed in the Supersomal enzymatic systems used here oxidized ellipticine only to three metabolites; 9-hydroxy-, 12-hydroxy-, and 13-hydroxyellipticine, again both in the presence of NADPH and NADH (Fig. 4). The formation of 7-hydroxyellipticine and the *N* ^2^-oxide of ellipticine was too low to be accurately quantified. NADH was less effective than NADPH as electron donor. In control incubations performed without the addition of external NADPH or NADH only low amounts of ellipticine metabolites were detected (Fig. 4).

Next, we utilized system (iii) where cytochrome *b*_5_ was over-expressed with CYP3A4 and POR at a molar ratio of CYP3A4:cytochrome *b*_5_ of 1:5 already in the cells from which CYP3A4-Supersomes™ (microsomes) were isolated. In the presence of NADPH, 4-, 5-, and 3.2-fold increased levels of 9-hydroxy-, 12-hydroxy-, and 13-hydroxyellipticine, respectively, were produced (Fig. 4). These results demonstrated that cytochrome *b*_5_ might function as a donor for the second electron transferred by POR from NADPH, which is in line with our earlier studies [15, 16]. In our previous work [15] a different procedure to include cytochrome *b*_5_ into Supersomes™ was utilized. Cytochrome *b*_5_ was reconstituted in Supersomes™, over-expressing POR with CYP3A4 in vitro (again in a molar ratio of CYP3A4:cytochrome *b*_5_ of 1:5). In this system, reconstitution with cytochrome *b*_5_ only increased the formation of 12-hydroxy- and 13-hydroxyellipticine but not that of 9-hydroxyellipticine [15]. This finding indicated that this heme protein does not only influence the transfer of electrons during the second reduction of CYP3A4, but that cytochrome *b*_5_ can also induce changes in the conformation of the CYP3A4 protein, leading to an altered profile of ellipticine metabolites [15]. Indeed, our previous studies have indicated a high specificity of interaction of CYP3A4 with holo-cytochrome *b*_5_ containing heme, which is necessary not only for electron transfer but also for the natural conformation of the cytochrome *b*_5_ and CYP3A4 proteins [15]. These results, and those found for the interaction of cytochrome *b*_5_ with CYP1A1 [17], demonstrate that the natural three-dimensional structure of the cytochrome *b*_5_ protein dictates the optimal conformational state of the CYP-cytochrome *b*_5_ complex. Further, the presence of the protoporphyrin IX bonded-Fe ion as an electron transfer agent is necessary for the observed effects.

Now, we can only speculate on the reasons for the different results found here and in our former studies [15]. One reason might be that the co-expression of the CYP3A4 and cytochrome *b*_5_ proteins from their *c*DNA into the membrane of endoplasmic reticulum may produce protein molecules in conformation states that are different from those generated after the reconstitution of cytochrome *b*_5_ with CYP3A4 in Supersomes™, thus leading to different enzymatic activity of the monooxygenase system. However, this suggestion awaits further investigation.

In the presence of NADH, cytochrome *b*_5_ over-expressed in CYP3A4-Supersomes™ also enhanced ellipticine oxidation to individual metabolites, but to different degrees; 9-hydroxyelliptice by 11-fold, 12-hydroxyellipticine by 1.3-fold, and 13-hydroxyellipticine by 6.5-fold, which led to the formation of an altered pattern of ellipticine metabolites compared to basal levels without over-expressed cytochrome *b*_5_ (Fig. 4). This finding demonstrated that cytochrome *b*_5_ influences the CYP3A4 catalytic activity in at least two ways. First, this heme protein can mediate CYP3A4 enzyme activity even in the absence of the NADPH/POR system, by donating both the first and second electron from NADH to CY3A4 in the catalytic cycle. This again indicates that the NADH:cytochrome *b*_5_ reductase as an NADH-dependent reductase can substitute POR in this enzymatic reaction and that the NADH/NADH:cytochrome *b*_5_/cytochrome *b*_5_ system might act as an exclusive donor of electrons for this CYP3A4-mediated ellipticine oxidation independent of NADPH and POR. Second, cytochrome *b*_5_ can also induce changes in the conformation of the CYP3A4 protein, resulting in changes in profile of ellipticine metabolite formation.

### Formation of ellipticine-derived-DNA adducts by human hepatic microsomes and human CYP3A4 expressed in Supersomes™ in the presence of NADPH and NADH

In further experiments, DNA adduct formation was analyzed in incubations with ellipticine and human hepatic microsomes or human CYP3A4 in Supersomes™ in the presence of DNA.

**Fig. 5:**
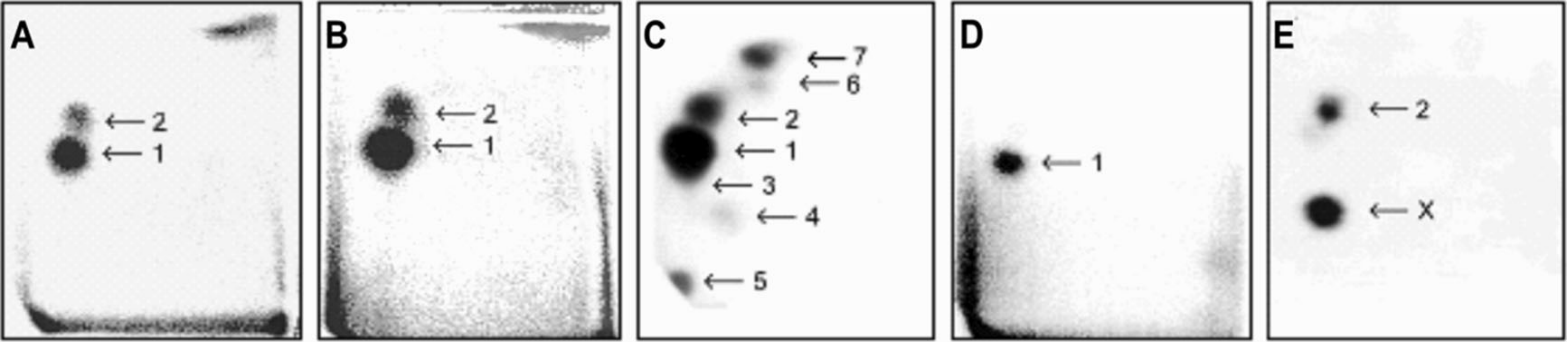
Autoradiographic profile of ^32^P-labeled DNA adducts generated in calf thymus DNA by ellipticine after its activation with CYP3A4 in the presence of NADPH without (**a**) and with cytochrome *b*_5_ (CYP3A4:cytochrome *b*_5_ of 1:5) (**b**): of ^32^P-labeled digests of DNA from the liver of a male rat treated with 40 mg ellipticine per kg body weight (**c**) [47]: from calf thymus DNA reacted with 13-hydroxyellipticine (**d**) [11] or 12-hydroxyelipticine (**e**) [12]. Analyses were performed by the nuclease P1 version of the ^32^P-postlabeling assay. Adduct spots 1–7 correspond to the ellipticine-derived DNA adducts

**Fig. 6:**
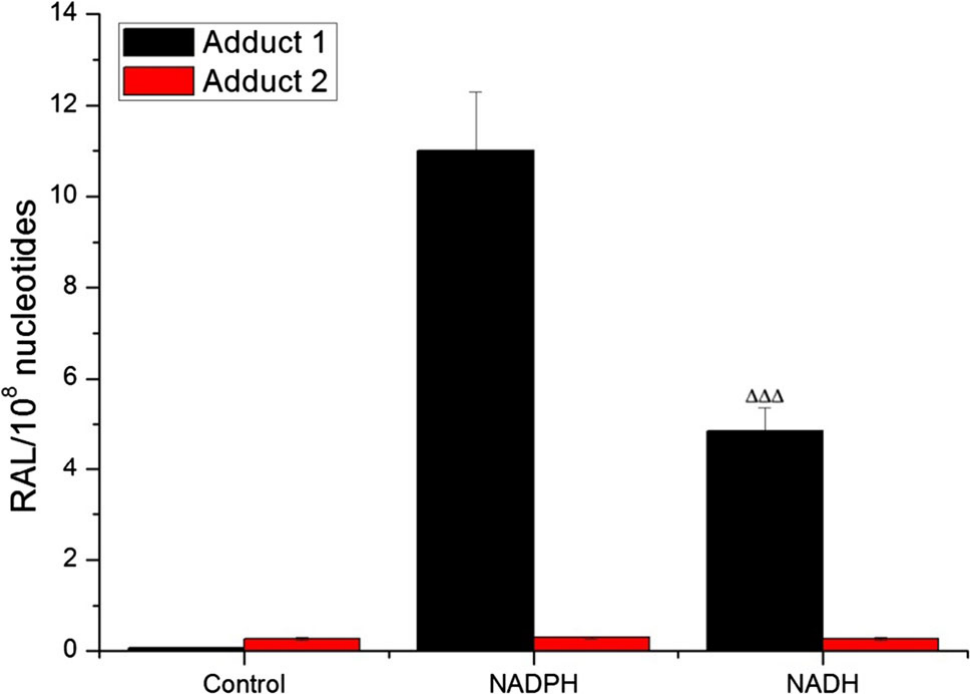
DNA adduct formation by ellipticine activated with human hepatic microsomes in the presence of either NADPH or NADH. Values represent the mean total RAL (relative adduct labeling) ± SD (*n* = 3; analyses of three independent in vitro incubations). ^∆∆∆^ *P* < 0.001 (Student’s *t* test), significantly different from incubations with NADPH. Control—incubations without added cofactors

**Fig. 7:**
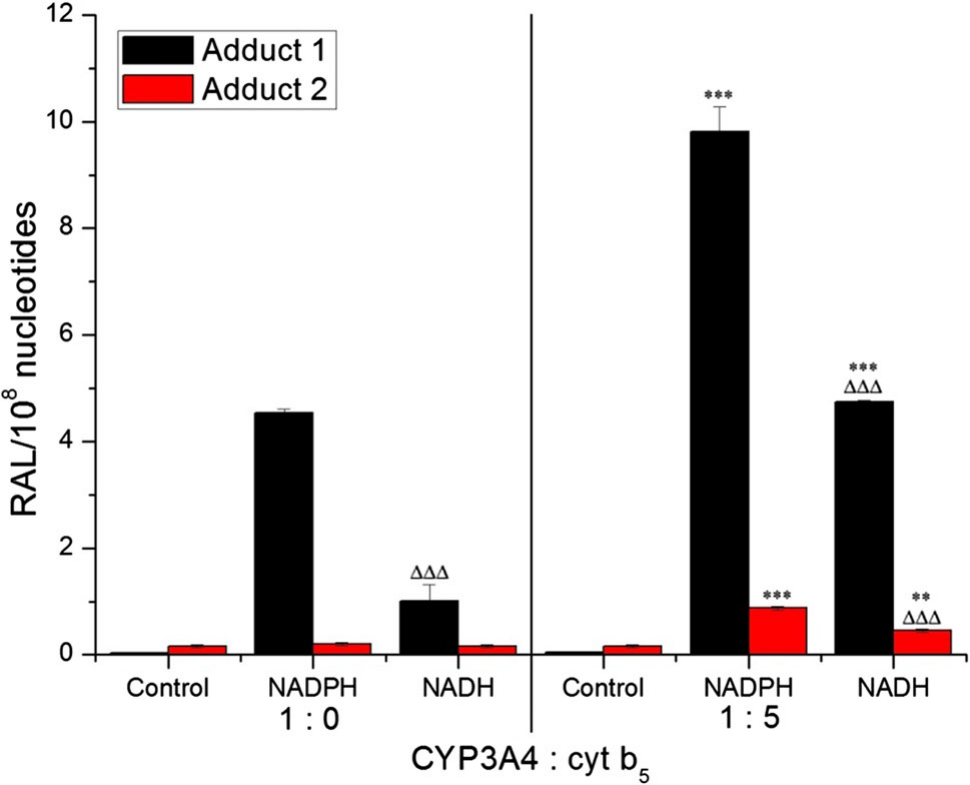
DNA adduct formation (RAL, relative adduct labelling) by ellipticine activated with human CYP3A4 in the presence of either NADPH or NADH and the effect of cytochrome *b*_5_ (cyt *b*_5_) (at a molar ratio of CYP3A4:cytochrome *b*_5_ of 1:5) on this reaction. Values represent mean total RAL (relative adduct labeling) ± SD (*n* = 3; analyses of three independent in vitro incubations). ^∆∆∆^ *P* < 0.001 (Student’s *t* test), levels of ellipticine-derived DNA adducts significantly different from incubations with NADPH as a cofactor; ***P < 0.001 (Student’s *t* test), levels of ellipticine-derived DNA adducts significantly different from incubations without cytochrome *b*_5_. Control—incubations without added cofactors

Two DNA adducts were detected by the ^32^P-postlabeling method [2, 11–16] derived from ellipticine activated with human hepatic microsomes or CYP3A4-Supersomes™ (Fig. 5), both in the presence of NADPH and NADH (Figs. 6, 7). One major adduct (assigned spot 1 in Fig. 5a–d) was previously shown [11] to be generated by the reaction of guanine in DNA with ellipticine-13-ylium which is formed by the decomposition of 13-hydroxyellipticine (Fig. 1). One minor adduct (assigned adduct spot 2 in Fig. 5a–c, e) was previously shown [12] to be generated by reaction of guanine in DNA with ellipticine-12-ylium formed from 12-hydroxyellipticine (Fig. 1). These two adducts were analogous to those formed in several cancer cells in vitro [43–46] and in healthy [13, 14, 47] (Fig. 5c) and tumor tissues in vivo [4].

The levels of ellipticine-derived adduct 1 (formed predominantly by CYP3A4) [11, 15, 16] generated in human microsomes were 2.8-fold lower with NADH than with NADPH as the cofactor (Fig. 6), which is in agreement with a 2.6-fold lower efficiency of ellipticine oxidation to 13-hydroxyellipticine by human microsomes in the presence of NADH (Fig. 2). Control incubations carried out without NADPH or NADH were essentially free of adduct spot 1, but adduct spot 2 was always detected (Fig. 6), indicating that adduct 2 might be also formed by autooxidation as reported previously [2, 4].

With human recombinant CYP3A4 expressed in Supersomes™ the adducts formed were the same as those generated by human hepatic microsomes, both in the presence of NADPH and NADH. But NADH as a coenzyme was again less efficient than NADPH (Fig. 7). When NADPH or NADH was omitted from the incubation mixtures containing CYP3A4-Supersomes™, only low amounts of adduct spot 2 were detected (Fig. 7).

In accordance with an increase in ellipticine oxidation catalyzed by CYP3A4-Supersomes™ with over-expressed cytochrome *b*_5_, shown in Fig. 4, the presence of this heme protein in the CYP3A4 system led to higher levels of ellipticine-derived DNA adducts, both in the presence of NADPH and NADH (Fig. 7).

## Conclusions

The present study was aimed to advance our knowledge regarding the role of cytochrome *b*_5_ in the reaction cycle of CYP3A4 catalyzing the oxidation of the anticancer drug ellipticine. Our results demonstrated that the function of cytochrome *b*_5_ in this reaction is complex. We found that oxidation of ellipticine by human CYP3A4 in the natural microsomal system of human liver or in Supersomes™, where the reductases POR and NADH:cytochrome *b*_5_ reductase and cytochrome *b*_5_ are expressed, is enhanced by cytochrome *b*_5_. Ellipticine oxidation does not only occur in the presence of the coenzymes of POR, NADPH, but also in the presence of NADH, which is the exclusive coenzyme of another microsomal reductase, NADH:cytochrome *b*_5_ reductase. These findings demonstrated that cytochrome *b*_5_ is included in the reduction of CYP3A4 catalyzed both by the NADPH/POR system and a system consisting exclusively of NADH, NADH:cytochrome *b*_5_ reductase and cytochrome *b*_5_, and thus also without any participation of NADPH and POR. Our data confirmed previous results found for CYP3A4 [26, 28] and for CYP1A1 [39, 40] that NADH:cytochrome *b*_5_ reductase/cytochrome *b*_5_/NADH can replace NADPH/POR in the catalytic cycle of the CYP reactions in the monooxygenase system [18, 19]. This is an important finding, because substitution of the POR/NADPH system by another reductase system might be clinically relevant for compensation or the lack of defect in POR in patients suffering from diseases caused by mutations of the *POR* gene. For example for patients with Antley-Bixler syndrome (OMIM 201750), a disorder characterized by severe developmental abnormalities and exhibiting considerable numbers of different haplotypes of the *POR* gene [48, 49]. Investigation of this feature is, therefore, a challenge for future studies.

Here, we showed that if NADPH is substituted by NADH, ellipticine oxidation is catalyzed by experimental CYP3A4 enzymatic systems. Such NADH-mediated activity of the employed CYP3A4 systems was proven by the formation of oxidized ellipticine metabolites as well as by the generation of ellipticine-derived DNA adducts, which were the same as in the system where NADPH was used as electron donor.

The results of our present study as well as those reported previously [15] indicated that cytochrome *b*_5_ can play a dual role in the CYP3A4-mediated oxidation of ellipticine. Specifically, as shown here, (i) it acts as a sole electron donor to CYP3A4 during its reaction cycle and (ii) it can also act as an allosteric modifier of CYP3A4 oxygenase [15]. The finding that cytochrome *b*_5_ with its reductase, NADH:cytochrome *b*_5_ reductase, functions as an exclusive donor for both electrons in the first and second reduction steps of the CYP3A4-catalyzed oxidation of ellipticine opens the door for further research. It will be a challenge to study the kinetics and structural features of this novel phenomenon.

## Experimental

### Chemicals and CYP3A4 subcellular systems

Ellipticine, NADH (as disodium salt; purity ~95%), and NADPH (as tetrasodium salt; ~98% purity) were obtained from Sigma Chemical Co (St Louis, MO, USA). Testosterone and 6-β-hydroxytestosterone were purchased from Merck (Darmstadt, Germany).

### CYP3A4 subcellular systems

CYP3A4-Supersomes™, microsomes isolated from insect cells transfected with a baculovirus construct containing cDNA of human CYP3A4 and POR that are, therefore, over-expressed in these microsomes, were purchased from the Gentest Corp. (Woburn, MI, USA). However, because they are microsomes (particles of broken endoplasmic reticulum), other enzymes (proteins) of the endoplasmic reticulum membrane (i.e. NADH:cytochrome *b*_5_ reductase and cytochrome *b*_5_) are also expressed at basal levels in these Supersomes™ (Gentest Corp., Woburn, MI, USA). We also utilized CYP3A4-Supersomes™ which also contained over-expressed cytochrome *b*_5_, in a molar ratio of CYP3A4 to cytochrome *b*_5_ of 1–5 (Gentest Corp., Woburn, MI, USA). Pooled human hepatic microsomes were from Gentest Corp. (Woburn, MI, USA).

### Incubations to study the metabolism of ellipticine

Incubation mixtures used to study the ellipticine metabolism by human hepatic microsomes or Supersomes™ contained in a final volume of 0.5 cm^3^ 100 mmol dm^−3^ potassium phosphate buffer (pH 7.4), 1 mmol dm^−3^ NADPH or NADH, 0.025 mmol dm^−3^ ellipticine (dissolved in 0.005 cm^3^ dimethyl sulfoxide, DMSO) and 0.5 mg protein of human hepatic microsomes or 100 nmol dm^−3^ human recombinant CYP3A4 in Supersomes™ either with or without cytochrome *b*_5_. The same amount of solvent (DMSO) was used in control incubations without ellipticine. With this DMSO concentration (1% final concentration), no inhibition of the NADPH-dependent CYP-catalyzed oxidation of several substrates has been found previously [10, 11, 15–17]. The reaction was initiated by adding the NADPH or NADH. Negative controls lacked either microsomes or Supersomes™ or cofactors or ellipticine. After incubation (37 °C, 20 min), 0.005 cm^3^ 1 mmol dm^−3^ phenacetin in methanol was added as an internal standard; ellipticine metabolism by microsomes has been shown to be linear up to 30 min of incubation [3, 11]. Ellipticine metabolites were extracted twice with ethyl acetate (2 × 1 cm^3^), the solvent was evaporated to dryness, residues were dissolved in 0.025 cm^3^ methanol and ellipticine metabolites were separated by HPLC as reported [3, 11, 15–17]. Ellipticine metabolite peaks were analyzed by HPLC by comparison with metabolite standards whose structures were determined previously by NMR and/or mass spectrometry [11, 12].

### Incubations to study 6-β-testosterone hydroxylation by CYP3A4 in Supersomes™

The incubation mixtures for measuring the testosterone metabolism contained in a final volume of 0.5 cm^3^: 100 mmol dm^−3^ potassium phosphate buffer (pH 7.4), 50 µmol dm^−3^ testosterone (0.0025 cm^3^ of stock methanol solution per incubation), 1 mmol dm^−3^ NADPH or NADH, and 100 nmol dm^−3^ human recombinant CYP3A4 in Supersomes™ either with or without cytochrome *b*_5_. The same amount of solvent (methanol) was used in control incubations without testosterone. The reaction was initiated by adding the NADPH or NADH. Negative control reactions lacked either CYP3A4-Supersomes™ systems, cofactors or testosterone. After incubation (for 15 min, at 37 °C in a shaking incubator), 0.005 cm^3^ 1 mmol dm^−3^ phenacetin in methanol was added as an internal standard; testosterone oxidation was linear up to 30 min of incubation (data not shown). The reaction was terminated by the addition of 0.1 cm^3^ of 1 mol dm^−3^ aqueous Na_2_CO_3_ containing 2 mol dm^−3^ NaCl. The metabolites were extracted with 2 × 1 cm^3^ of dichloromethane and the extracts were evaporated to dryness. The residues were dissolved in the mobile phase for HPLC (see below). Testosterone and its metabolite 6-β-hydroxytestosterone were separated on Nucleosil (C18) HPLC column (4.6 × 25 mm, 5 μm, Macherey–Nagel, Germany). The flow rates, mobile phases and detection wavelength were 0.7 cm^3^ per min, 65:35 methanol/H_2_O (v/v), and 254 nm, respectively [50, 51].

### Determination of DNA adduct formation by ellipticine by ^32^P-postlabeling

Incubation mixtures used to assess DNA adduct formation by ellipticine contained 0.5 mg protein of human hepatic microsomes or 100 nmol dm^−3^ human recombinant CYP3A4 in Supersomes™ either with or without cytochrome *b*_5_, 0.1 mmol dm^−3^ ellipticine (dissolved in 0.0075 cm^3^ methanol), and 0.5 mg of calf thymus DNA in a final volume of 0.75 cm^3^ as described previously [2, 4, 15, 16]. The reaction was initiated by adding 0.1 mmol dm^−3^ ellipticine and incubations were carried out at 37 °C for 60 min. The ellipticine-derived DNA adduct formation was shown to be linear up to 90 min [2, 11, 15, 16]. Control incubations were carried out either without human hepatic microsomes or CYP3A4 in Supersomes™ without NADPH (or NADH), without DNA, or without ellipticine. After the incubation, DNA was isolated from the residual water phase by standard phenol/chloroform extraction. DNA adduct formation was analysed using the nuclease P1 version of the ^32^P-postlabeling technique [2, 11, 15, 16]. Resolution of the adducts by thin-layer chromatography (TLC) using polyethylenimine-cellulose plates (Macherey and Nagel, Düren, Germany) was carried out as described [2, 11, 15, 16]. DNA adduct levels (*RAL* relative adduct labeling) were calculated as described [52].

### Statistical analyses

Statistical analyses were carried out with mean ± standard deviations (from the original data) of at least three parallel experiments with Student’s *t* test (UNISTAT Statistics Software v6, Unistat Ltd., Highgate, London N6 5UQ, UK). All *P* values were two-tailed and considered significant at the 0.05 level.
